# Changes of Vision-Related Quality of Life in Retinal Detachment Patients after Cataract Surgery

**DOI:** 10.1371/journal.pone.0120505

**Published:** 2015-03-12

**Authors:** Mingming Zhu, Jiannan Huang, Bijun Zhu, Qian Sun, Xian Xu, Yuyu Miao, Haidong Zou

**Affiliations:** 1 Department of Ophthalmology, Shanghai First People’s Hospital, Shanghai JiaoTong University, Shanghai, China; 2 Shanghai Eye Disease Prevention & Treatment Center, Shanghai, China; Medical College of Soochow University, CHINA

## Abstract

Rhegmatenous retinal detachment (RRD) is one of the most serious complications after phacoemulsification combined with intraocular lens implantation surgery. It has been reported that vision-related quality of life (VRQoL), as well as visual acuity rapidly decreased when RRD developed. However, little is known of the VRQoL in those RRD patients after anatomical retinal re-attachment, especially whether or not the VRQoL is higher than that before cataract surgery. In this prospective case series study, we use the Chinese-version low vision quality of life questionnaire (CLVQOL) to assess the changes of VRQoL in age-related cataract patients who suffered from RRD after phacoemulsification with intraocular lens (phaco-IOL) implantation. All participants were asked to complete questionnaires in face- to-face interviews one day before and two weeks after cataract surgery, as well as one day before and three months after RRD surgery. A total of 10,127 consecutive age-related cataract patients were followed up to one year after phaco-IOL implantation; among these patients, 17 were diagnosed as RRD. The total CLVQOL scores and subscale scores except “Mobility” decreased significantly when RRD developed. After retinal surgery, only the score of “General vision and lighting” in the CLVQOL questionnaires improved when compared to the scores two weeks after cataract surgery, although the best corrected visual acuity of all patients significantly raised up. However, the mean CLVQOL scores and subscale scores were still considerably higher than the level prior to cataract surgery. Our study suggests that cataract patients at high risk of postoperative RRD should not deny the opportunity to undergo phaco-IOL implantation, even though potential VRQoL impairment induced by RRD exists.

## Introduction

Currently, phacoemulsification combined with intraocular lens implantation surgery (hereinafter referred to as “phaco-IOL”) is the most popular and effective treatment for cataract. Its advantages include less injury, rapid vision recovery, and satisfactory curative effect. Numerous studies have shown that the vision-related quality of life (VRQoL) of cataract patients was significantly improved after phaco-IOL [1–4]. However, it is still inevitable to suffer vision loss caused by phaco-IOL-related complications, one of which is rhegmatenous retinal detachment (RRD). It has been reported that the VRQoL, along with visual acuity (VA) rapidly decreased as soon as RRD developed, even though the retina was re-attached after surgery [5]. It has been estimated that the 5-year cumulated incidence of RRD after phaco-IOL varies from 0.67% to 0.71% [6,7]. The reported risk factors of RRD in pseudophakic eyes include long axial lengths, posterior capsule rupture, neodymium: yttrium-aluminum-garnet laser capsulectomy, and more [8–11]. For example, the incidence of RRD was 1.5% to 2.2% in pseudophakic eyes with an axial length of 27.0 mm or more [12]. Therefore, the cataract patients at high risk of postoperative RRD will inevitably reconsider the choice of phaco-IOL after preoperative communication with surgeons.

Questionnaires of quality of life focused on vision are generally used to evaluate and quantify the influence of ocular diseases on daily life and changes made by treatment or surgery [13,14]. A study involving 101 eyes after uncomplicated extracapsular cataract extraction (ECCE) conducted with a tool of the visual functioning index VF-14 showed that recovery of anatomic and functional visual outcome in pseudophakic RRD patients was limited when they are reading small prints, watching steps, and undertaking fine works. However, the VRQoL in RRD patients after phaco-IOL is still known little [15], and it it is still unknown whether or not the VRQoL after anatomical retinal re-attachment is higher than that before cataract surgery. The answer to this question, to a certain extent, will help the patients in high risk of pseudophakic RRD to make their decision for cataract surgery. For this reason, we conducted this study to investigate changes of VRQoL in pseudophakic RRD patients by evaluating patients who received phaco-IOL in the Department of Ophthalmology, Shanghai First People’s Hospital, Shanghai Jiao Tong University, Shanghai, China, from 2005 to 2007.

## Materials and Methods

We used a Chinese-version low vision quality of life questionnaire (CLVQOL), originally translated from the English-language Low Vision Quality of Life Questionnaire (LVQOL), to assess the changes of VRQoL in RRD patients, this approach has been used previously [16,17]. This questionnaire includes 25 close-ended items which are all graded on an ordinal scale between 5 (no problem due to vision) and 1 (great difficulty due to vision). These items can also be scored as no longer possible due to vision (attributed a grade of 0) or as not relevant to them in their daily lives (attributed the average score of their total responses to avoid bias in the results of those who had less items relevant to them than others). A summed CLVQOL score ranged from 0 (representing binocular no light perception (NLP)) to 125 (representing best vision function). The VARIMAX rotation factor analysis identified four principal factors (eigenvalues over 1), of the CLVQOL, in which the 25 items were then grouped into four subscales: general vision and lighting (GL), from item 1 to 7; mobility (M): from item 8 to 12; psychological adjustment (PA): from item 13 to 16; and reading, fine work and activities of daily living (RFA): from item 17 to 25.

Inclusion criteria included: Chinese speaking, neither debilitated nor with psychiatric disorders, and RRD patients who had received phaco-IOL in the Department of Ophthalmology, Shanghai First People’s Hospital, Shanghai Jiao Tong University, Shanghai, China, from Jan 1^st^, 2005 to Dec 31^st^, 2007.

Prior to cataract surgery, all patients were informed about RRD symptoms, such as sudden vision loss or a “shadow” or “curtain” (scotoma) spreading across the field of vision, and were asked to come back immediately for further treatment in case of RRD symptoms happen. They completed the CLVQOL by themselves or with the help of trained investigator one day before and two weeks after phaco-IOL, one day before and three months after RRD surgery.

A well skilled interviewer helped the patients during face-to-face inquiry, if necessary. The collected data included general information for the patients (e.g, name, gender, age, education level, etc.); eye information (e.g, refraction, best corrected VA (BCVA) in each eye, proliferative vitreoretinopathy grade (according to the 1983 Retinal Society) [18], retina quadrants detached in extent, and macular detachment, etc,); and surgical information (e.g, surgical procedures, intraoperative and postoperative complications, etc,). The surgical procedures were classified as (1) scleral buckling surgery: cryopexy, circumferential silicone band encircling, and subretinal fluid drainage when required; (2) vitreous surgery: vitrectomy, membranectomy, internal drainage of the subretinal fluid, endophotocoagulation, injection of gas, related procedures, or combined with scleral buckling surgery.

All of the enrolled participants provided written informed consent for participation in this study. This study was conducted according to the tenets of the Declaration of Helsinki, and was approved by Ethics Committee on Human Research of Shanghai First People's Hospital, Shanghai Jiao Tong University.

The VA and CLVQOL scores (e.g, item, subscale composite and total) were compared between four research phases: one-day before phaco-IOL as phase 1 (P1), two-week after phaco-IOL as phase 2 (P2), one-day before RRD surgery as phase 3 (P3), and three-months after RRD surgery as phase 4 (P4). The Snellen fractions were converted to a log scale using the method of Holladay and Prager [19, 20]. Weighted average LogMAR vision represents a summary score of VA encompassing visual information from both eyes, with the better eye given a weight of 0.75 and the worse eye a weight of 0.25 [21]. Since all LogMAR VA values in each research phase, no matter in surgery eye or weighted average, follow Gaussian distribution according to one-sample Kolmogorov Smirnov test (Z ranged from 0.45 to 1.22, all *P*>0.05), they were presented as mean values and standard deviations. The paired *t*-test was used in the comparisons of LogMAR VA between different research phases.

We used the Cronbach’s α coefficient to estimate the reliability of CLVQOL in Chinese RRD patients. The ordinal CLVQOL item, subscale composite and total scores were presented as median and range. Mann Whitney U test of nonparametric tests were used in the comparison of CLVQOL scores between different research phases. Bivariate Spearman correlation analyses were performed to determine the associations between the CLVQOL composite score at the end of follow-up time (three-months after RRD surgery) and the variables of interest (socio-demographic and clinical characteristics). A *P* value less than 0.05 was considered statistically significant (SPSS v17.0, Chicago, Illinois). To further assess the influence of RRD on the VRQoL of phaco-IOL patients, the “Score decrement” was calculated by the following equation: [(MSC − MSR) / MSC] × 100%, MSC represents mean score of the Post-phaco-IOL group, and MSR represents mean score of Pre- Retinal Detachment Surgery group.

## Results

Among the 10,127 recruited patients, 17 patients were diagnosed as RRD in one-year postoperatively, giving an index of risk of RRD in pseudophakic patients as 0.17%. The age of the 17 RRD patients ranged from 50 to 76 years, with a mean of 63 years (Standard deviation, SD = 7.99). None of the patients was suffering from systemic co-morbidities or other ocular disorders. All of the patients were monocular RRD patients, and RRD developed from four-weeks to 31-weeks after phaco-IOL, with a mean time of 20.29 weeks (SD = 8.09). The socio-demographic and clinical characteristics of the 17 patients are listed in Table 1.

**Table 1:** Socio-demographic and clinical characteristics of the 17 patients with RRD in the present study.

	Number (%)
**Male**	9(52.94)
**Age, years**	
< = 60	7(41.18)
>60	10(58.82)
**Education, years**	
< 9	1(5.88)
9–12	9(52.94)
> = 12	7(41.18)
**Snellen BCVA in the RRD eye**	
20/60–20/20	3(17.65)
20/400–20/60	10(58.82)
<20/400	4(23.53)
**Refractions of the RRD eye before cataract surgery**	
highly myopic (< −6D)	6(35.29)
myopic (−6D to −0.5D)	8(47.06)
emmetropic or hyperopic (> −0.5D)	3(17.65)
**Complications of phaco-IOL**	
posterior capsule rupture	6(35.29)
None	11(64.71)
**Time gap between phaco-IOL and RRD**	
< 12 week	3(17.65)
12–24 weeks	8(47.06)
> 24 weeks	6(35.29)
**Proliferative vitreoretinopathy grade**	
A, B	4(23.53)
C, D	13(76.47)
more than 2 quadrants detached in RRD eyes	12(70.59)
macula detachment in RRD eyes	14(82.35)
**Time gap between RRD onset and RRD surgery**	
within 2 days	11(64.71)
more than 2 days	6(35.29)
**RRD surgical procedures**	
scleral buckling surgery	2(11.76)
vitreous surgery	15(88.24)
**Complications of RRD surgery**	8(47.06)

BCVA: best corrected visual acuity; RRD: rhegmatogenous retinal detachment

Preoperative and postoperative LogMAR BCVA in the 17 pseudophakic RRD patients is shown in Table 2. BCVA was obviously improved and mostly better than 0.50 after phaco-IOL. When RRD developed, BCVA of RRD eyes decreased rapidly to the pre-phaco-IOL level and did not return to post-phaco-IOL level after all retinas re-attached after vitreous or scleral buckling surgery. However, while comparing to pre-phaco-IOL level, the final BCVA was significantly better.

**Table 2:** BCVA in 4 research phases of 17 RRD patients in the present study.

	Mean ± Standard Deviation				Statistic value t ƚ					
	P1	P2	P3	P4	P1:P2	P2:P3	P3:P4	P1:P3	P1:P4	P2:P4
In the RRD Eyes	1.21 ± 0.47	0.41 ± 0.34	1.17 ± 0.56	0.81 ± 0.22	10.27^§^	−5.20^§^	3.07^§^	0.25 P = 0.81	2.91^§^	−3.50^§^
Weighted average	0.57 ± 0.17	0.39 ± 0.16	0.56 ± 0.21	0.47 ± 0.14	8.33^§^	−4.92^§^	3.06^§^	0.24 P = 0.81	2.91^§^	−3.23^§^

BCVA: best corrected visual acuity, RRD: rhegmatogenous retinal detachment, 4 research phases: one-day before phaco-IOL as phase 1 (P1), 2-week after phaco-IOL as phase 2 (P2), one-day before RRD surgery as phase 3 (P3), and 3-month after RRD surgery as phase 4 (P4)

ƚ Paired t-test

§ P<0.05

Of the 17 participants, the Cronbach coefficients of CLVQOLs in the pre-phaco-IOL, post-phaco-IOL, pre-RRD surgery and post-RRD surgery time points were 0.92, 0.91, 0.90, and 0.92, respectively. The CLVQOL scores are shown in Table 3. The composite scores of subscales GL, M, PA, RFA, and total CLVQOL improved significantly after phaco-IOL as compared to those before phaco-IOL. When RRD developed, the subscale GL, PA, RFA, and total CLVQOL scores decreased significantly. The score decrement (%) in GL, M, PA and RFA subscales was 28.13%, 8.3%, 31.25%, and 16.67%, respectively. Further analysis in items indicated that there was no significant reduction in items of “getting around outdoors”, “crossing a road with traffic”, “vision in general” in M subscales, “unhappy at the situation in life” in PA subscale, “reading your letters and mail”, “having problems using tools”, “reading own hand writing”, and “every day activities” in RFA subscale (all *P*>0.05).

**Table 3:** CLVQOL scores in 4 research phases in 17 pseudophakic RRD patients.

	Median (range)				Statistic value Z ƚ			
	P1	P2	P3	P4	P1:P2	P2:P3	P3:P4	P1:P4
**General vision and lighting**	17(10–23)	32(25–35)	23(14–34)	27(13–15)	−5.00^§^	−3.81^§^	−2.13^§^	−4.47^§^
when eyes getting tired	2(1–3)	5(3–5)	3(2–5)	4(2–5)	−5.07^§^	−4.00^§^	−2.40^§^	−4.69^§^
vision at night inside the house	2(1–3)	5(3–5)	3(2–5)	4(2–5)	−4.99^§^	−3.62^§^	−2.73^§^	−4.81^§^
when getting the right amount of light to be able to see	3(1–5)	5(3–5)	3(2–5)	4(2–5)	−3.19^§^	−2.45^§^	−1.88	−2.80^§^
poblems with glare	2(1–3)	4(3–5)	3(2–5)	4(2–5)	−4.69^§^	−2.07^§^	−2.40^§^	−4.72^§^
seeing street signs	2(1–5)	5(3–5)	3(2–4)	4(1–5)	−4.22^§^	−3.95^§^	−1.74	−2.18^§^
seeing the television	2(1–4)	5(3–5)	4(1–5)	4(1–5)	−5.03^§^	−3.20^§^	−0.07	−2.85^§^
seeing moving objects	3(1–5)	5(4–5)	3(1–5)	4(3–5)	−4.85^§^	−3.91^§^	−2.15^§^	−3.63^§^
**Mobility**	15(9–24)	24(18–25)	22(15–25)	21(13–25)	−4.04^§^	−1.61	−0.05	−2.68^§^
judging the depth or distance of items	4(1–5)	5(4–5)	5(2–5)	4(3–5)	−2.80^§^	−2.36^§^	0.39	−0.80
seeing steps or curbs	3(1–5)	5(4–5)	4(2–5)	5(3–5)	−4.14^§^	−3.27^§^	−0.90	−2.45^§^
getting around outdoors	3(2–5)	5(3–5)	4(2–5)	4(3–5)	−3.11^§^	−1.58	−0.02	−2.34^§^
crossing a road with traffic	3(1–5)	5(3–5)	5(2–5)	4(3–5)	−3.11^§^	−1.20	−0.15	−2.37^§^
vision in general	2(1–4)	4(3–5)	4(3–5)	4(1–5)	−4.30^§^	−0.23	−1.62	−3.52^§^
**Psychological adjustment**	10(5–14)	16(12–20)	11(6–20)	14(11–20)	−4.55^§^	−3.26^§^	−1.95	−4.50^§^
unhappy at the situation in life	3(1–3)	4(3–5)	4(2–5)	3(2–5)	−4.79^§^	−1.80	−1.47	−2.91^§^
frustrated at not being able to do certain tasks	3(1–3)	4(2–5)	3(1–5)	3(1–5)	−4.14^§^	−3.27^§^	−1.28	−2.80^§^
restricted in visiting friends or family	2(1–3)	4(2–5)	3(1–5)	3(2–5)	−4.33^§^	−3.42^§^	−2.19^§^	−3.81^§^
how well the eye condition has been explained	3(1–5)	4(3–5)	3(1–5)	5(3–5)	−3.35^§^	−3.25^§^	−4.07^§^	−4.16^§^
**Reading, fine work and activities of daily living**	14(4–20)	42(28–45)	35(23–41)	35(25–42)	−4.99^§^	−3.31^§^	−0.44	−4.99^§^
reading large print	3(1–5)	5(3–5)	3(1–5)	4(3–5)	−3.66^§^	−3.95^§^	−1.94	−1.52
reading newspaper and books	3(1–5)	5(3–5)	4(2–5)	3(3–5)	−4.38^§^	−2.8°^§^	−2.80^§^	−2.14^§^
reading labels	2(1–5)	4(3–5)	3(2–5)	3(3–5)	−3.85^§^	−2.55^§^	−0.74	−2.56^§^
reading your letters and mail	3(1–5)	4(2–5)	3(2–5)	3(3–5)	−3.00^§^	−1.35	−0.90	−2.27^§^
having problems using tools	3(1–5)	4(1–5)	3(2–5)	4(1–5)	−2.98^§^	−1.69	−1.65	−3.15^§^
finding out the time	3(1–5)	5(4–5)	5(2–5)	3(2–5)	−4.13^§^	−2.76^§^	−1.55	−0.38
writing	3(1–5)	5(3–5)	4(1–5)	4(3–5)	−3.71^§^	−3.38^§^	−0.31	−0.66
reading own hand writing	3(1–5)	5(3–5)	4(3–5)	4(3–5)	−2.12^§^	−0.84	−1.21	−0.92
every day activities	3(1–5)	5(2–5)	4(3–5)	4(3–5)	−2.96^§^	−1.20	−0.81	−2.36^§^
**Total**	71 (34–96)	114 (84–125)	89 (67–119)	98 (62–115)	−4.88^§^	−3.81^§^	−1.23	−4.09^§^

CLVQOL: Chinese-version low vision quality of life questionnaire, RRD: rhegmatogenous retinal detachment 4 research phases: one-day before phaco-IOL as phase 1 (P1), 2-week after phaco-IOL as phase 2 (P2), one-day before RRD surgery as phase 3 (P3), and 3-month after RRD surgery as phase 4 (P4)

ƚ Mann Whitney U test

§ P<0.05

As shown in Table 3, compared to pre-RRD surgery status, no significant increase was found in scores of subscale M, PA, PFA, and total CLVQOL three-months after retina re-attachment. The improvement of scores was only found in the following items: “when eyes getting tired”, “vision at night inside the house”, “problems with glare”, “problems with seeing moving objects”, in GL subscale; “restricted in visiting friends or family”, “how well the eye condition has been explained” in PA subscale; and “reading newspaper and books” in RFA subscale (all *P*<0.05). Nevertheless, compared to scores before phaco-IOL, most of the item scores after retina surgery were significantly higher.

After bivariate correlation analyses, a higher total CLVQOL composite score in three-months after RRD surgery was associated with female gender (Spearman correlation, *r* = 0.59, *P*<0.05), younger age (Spearman correlation, *r* = 0.56, *P*<0.05), and no postoperative complications (Spearman correlation, *r* = 0.82, *P*<0.05). No statistical significant correlation was found between the total CLVQOL composite score and education time, refraction, weighted average BCVA, BCVA in RRD eye, proliferative vitreoretinopathy grade, retinal quadrants detached, and macular detachment.

## Discussion

It has been proved that CLVQOL can satisfy conventional psychometric criteria, identify low vision populations and benefits of low vision rehabilitation [16]. In this study, we show a high Cronbach’s α coefficient, which is similar to what we previously calculated in RRD patients [22,23], and further indicates that this measure has acceptable reliability in pseudophakic RRD patients.

In this study and similar to other studies, cataract surgery extremely improved VRQoL of cataract patients [1–4].However, when RRD developed we found that the VRQoL estimated by CLVQOL decreased sharply. In one of our previous studies, we confirmed that comparing to non-RRD subjects, a score decrement (15.12%) of CLVQOL in subscale GL happened in the general RRD patients [22]. In this study, the score decrement (28.13%) was much higher and may be attributed to different study subjects observed. However, similar to our previous work [22], this study proved that the scores of VRQoL items related to stereoscopic vision, “when getting the right amount of light to be able to see” and “seeing moving objects”, were minimally affected by RRD in pseudophakic patients, and the lowest reduction ratio was found in subscale M.

In another previous study made by our group, we made a CLVQOL assessment on 92 general RRD patients who were followed for three years after RRD surgery, and found that statistically significant CLVQOL score increase occurred in all the composite scores of four subscales in the three-month postoperative time [23]. However in this study, even though the BCVA of RRD eyes was significantly improved when the retina was re-attached, significant positive score changes were only found in subscale GL. We consider that the different study design may contribute to the different pattern of VRQoL recovery between the two studies. For instance, in this study, the proportion of RRD patients with macular detachment (82.35%) was much higher than that in previous study (65.2%) [23]. Our previous work proved that a higher three-year postoperative CLVQOL total score was associated with preoperative macula-on in RRD patients. Therefore, it is plausible that the scores of subscales M and RFA, which to a great extent depend on central vision, did not significantly increase three months after RRD surgery in a majority of the 17 patients in the present study. On the contrary, the scores of some items, such as “vision at night inside the house” and “Problems with seeing moving objects” in subscale GL, which correlate mainly to stereopsis or scotopic vision, increased significantly in the first three months postoperatively.

The influences of RRD on patients were found not only on functional aspect but also psychology level. Compared to CLVQOL scores after phaco-IOL surgery, the highest subscale score decrement (31.25%) was found in PA, which was similar with the result reported in our previous study [22]. It is understandable that a sudden decrease in VA would cause serious panic to patients. It’s worth noting that the influence on psychology level did not disappear within three-months after RRD surgery, even the BCVA was significantly improved. Those results indicated that more attention should be paid to the influences of RRD on psychology of patients.

Postoperative complications will definitely influence the early recovery of VRQoL. Therefore, it is reasonable that no complications were one of the relative factors resulting in better three-month postoperative VRQoL. For some unknown reason, female and younger patients both correlate with final high CLVQOL scores. More lines of evidences in the future will help to prove that this association is causal rather than coincidental. An exciting finding of this study was that VRQoL of pseudophakic RRD patients after retinal surgery was much better than that before they received phaco-IOL. Since most of the RRD patients lost the long-term follow-up, we only reported the VRQoL change three months after RRD surgery. Because scores of CLVQOL may keep rising up to a stable level until one year after retinal surgery [23], we have reason to surmise that final VRQoL of pseudophakic RRD patients might be even better than the level that we assessed after surgery. Thus, it is suggested that cataract patients who were with high-risk factors should not deny their chance to undergo phaco-IOL, if for no other reason than the VRQoL impairment due to probable RRD. Furthermore, as long as the retinal was re-attached, pseudophakic RRD patient can still gain larger improvement of their quality of life.

One limitation in this study is the small sample size. We followed up 10,127 cataract patients, but a very low occurrence of RRD after cataract surgery attributes to this inherent defect of this study. In addition, as a result of high loss follow-up ratio, we are not able to obtain a longer-term data of pseudophakic RRD patients after retinal surgery. Perhaps because small amount of patients were enrolled, we find some unreasonable changes in items of “reading your letters and mail”, “having problems using tools”, “reading own hand writing”, or “every day activities” when retinal detachment developed and items of “When eyes getting tired”, “Problems with glare”, or “reading newspaper and books” when retina was re-attached. Also for this reason, we did not observe statistically significant relationships between BCVA and CLVQOL scores. In our previous study, it was reported that a higher three-year postoperative CLVQOL composite score was associated well with a better three-year postoperative weighted average BCVA in the general RRD patients [23]. We suggest using more subjects that are in a longer-term postoperative status in future work to help further elucidate the VRQoL change and related factors in pseudophakic RRD patients.

## Conclusions

RRD, which can cause sudden vision loss and VRQoL reduction, has been a scruple to some cataract patients who have high myopia or some other eye diseases. In this study, we found that although the VRQOL scores decrease when RRD occurred, patients could have better QOL than that of pre-phaco-IOL level. This finding will give those cataract patients who have higher risk of RRD more confidence to receive phacoemulsification.
